# High Incidence of Related *Wolbachia* across Unrelated Leaf-Mining Diptera

**DOI:** 10.3390/insects12090788

**Published:** 2021-09-03

**Authors:** Xuefen Xu, Peter M. Ridland, Paul A. Umina, Alex Gill, Perran A. Ross, Elia Pirtle, Ary A. Hoffmann

**Affiliations:** 1 Bio21 Institute, School of BioSciences, The University of Melbourne, Parkville, VIC 3010, Australia; p.ridland@unimelb.edu.au (P.M.R.); pumina@unimelb.edu.au (P.A.U.); alex.gill@unimelb.edu.au (A.G.); perran.ross@unimelb.edu.au (P.A.R.); 2 Cesar Australia, 95 Albert St, Brunswick, VIC 3056, Australia; epirtle@cesaraustralia.com

**Keywords:** leaf-mining diptera, agromyzidae, *Wolbachia*, *wsp*, MLST, cytoplasmic incompatibility

## Abstract

**Simple Summary:**

Polyphagous leaf-mining flies of the genus *Liriomyza* are pests that pose a serious threat to agricultural and horticultural industries. The endosymbiotic bacterium *Wolbachia* has been proposed as a useful biocontrol strategy for managing pests, but few studies have so far examined *Wolbachia* in leafminers. We find a high incidence of related *Wolbachia* in a survey of infections in 13 dipteran leafminer species collected from Australia and elsewhere which could potentially be useful for the incompatible insect technique (IIT) of pest suppression. We performed curing and crossing experiments on *L. brassicae* to demonstrate the presence of cytoplasmic incompatibility (CI) needed for IIT, providing a foundation for future transfection of CI *Wolbachia* from *L. brassicae* to other *Liriomyza* pests. Overall, these findings highlight a high incidence of *Wolbachia* in leaf-mining Diptera, potential horizontal transmission events and possible applications of *Wolbachia*-based biocontrol strategies for *Liriomyza* pests.

**Abstract:**

The maternally inherited endosymbiont, *Wolbachia pipientis,* plays an important role in the ecology and evolution of many of its hosts by affecting host reproduction and fitness. Here, we investigated 13 dipteran leaf-mining species to characterize *Wolbachia* infections and the potential for this endosymbiont in biocontrol. *Wolbachia* infections were present in 12 species, including 10 species where the *Wolbachia* infection was at or near fixation. A comparison of *Wolbachia* relatedness based on the *wsp*/MLST gene set showed that unrelated leaf-mining species often shared similar *Wolbachia*, suggesting common horizontal transfer. We established a colony of *Liriomyza brassicae* and found adult *Wolbachia* density was stable; although *Wolbachia* density differed between the sexes, with females having a 20-fold higher density than males. *Wolbachia* density increased during *L. brassicae* development, with higher densities in pupae than larvae. We removed *Wolbachia* using tetracycline and performed reciprocal crosses between *Wolbachia*-infected and uninfected individuals. Cured females crossed with infected males failed to produce offspring, indicating that *Wolbachia* induced complete cytoplasmic incompatibility in *L. brassicae*. The results highlight the potential of *Wolbachia* to suppress *Liriomyza* pests based on approaches such as the incompatible insect technique, where infected males are released into populations lacking *Wolbachia* or with a different incompatible infection.

## 1. Introduction

The genus *Liriomyza* is one of the most widely studied and well-documented groups in the Agromyzidae. Since the 1990s, three polyphagous species-*Liriomyza huidobrensis* (Blanchard), *Liriomyza trifolii* (Burgess) and *Liriomyza sativae* Blanchard-have colonized many new areas around the globe [1,2], likely through an increasing international trade in vegetable and horticultural products which has led to their spread on infested plant material [3]. Introduced leaf-mining pests are prone to outbreaks and rapidly become uncontrollable, which has allowed the establishment of these species in most countries [3,4,5,6,7]. This includes Australia, where *L. sativae* and *L. huidobrensis* have become established pests and *L. trifolii* has recently invaded, posing a significant economic threat to Australian agricultural and horticultural industries [8,9,10].

Adults and larvae of *Liriomyza* flies cause damage to host plants. Female flies damage plants by puncturing the epidermis of host plant leaves with their ovipositor for feeding and egg-laying [11,12]. The leaf punctures also provide entry sites for plant pathogenic bacteria and fungi [13,14,15]. Males and female flies feed on the exudates from the punctures made by females [11]. Most damage is caused by the larval stage tunneling through the palisade and spongy mesophyll cells, producing serpentine mines and reducing the photosynthetic capacity of plants [16], with severely infested leaves falling off plants.

*Liriomyza* spp. are classic secondary pests, triggered by pest management approaches to control leafminer pests, which routinely rely on insecticide applications [17,18]. Indiscriminate use of broad-spectrum insecticides such as methomyl, methamidophos and permethrin has led to adults evolving insecticide resistance [19,20,21], while larvae are inaccessible to many insecticides because they are embedded in the foliar tissue and pupate in soil [12,22]. There is a higher activity of detoxification enzymes in larvae in comparison to adults, leading to rapid detoxification or sequestration of insecticides [23]. Translaminar insecticides such as abamectin and cyromazine can provide effective chemical control as they penetrate the leaves and kill larvae [24,25]. However, these insecticides have been reported to impact beneficial parasitoid populations [24], they are more expensive than older broad-spectrum insecticides and resistance to these chemicals has been documented [20,21].

Biological control is a safe and sustainable approach that exploits natural enemies (microorganisms, parasitoids, predators and pathogens) to reduce or suppress pest populations [26,27,28]. Augmentative releases of the eulophid parasitoid, *Diglyphus isaea* (Walker), are widely used for *Liriomyza* control in ornamental and vegetable greenhouses worldwide [29,30]. The system works well in many vegetable crops because *Liriomyza* spp. do not attack the harvested produce, and it can also be used successfully for some ornamental crops where mined lower leaves are removed at harvest [30]. In these cases, early releases of parasitoids prevent mining on the upper leaves close to the flowers [30]. Despite this, it can be difficult to establish *D. isaea* in winter in greenhouses when growth lights attract and kill adult parasitoids [31]. Another challenge is that the sex ratios of commercially reared *D. isaea* may be extremely male-biased (up to 77% male) [32], resulting in high costs which can make augmentative biological control of leafminers expensive [33,34]. This has led to interest in developing additional control strategies. For instance, releases of sterile *L. trifolii* males could be undertaken in combination with releases of *D. isaea,* with synergistic effects suggested in trials on potted chrysanthemums [35].

The incompatible insect technique (IIT) involves endosymbiont-induced cytoplasmic incompatibility (CI) when males with an infection mate with females lacking the infection and cause embryonic mortality in filial generations; the CI generated from the repeated release of infected males then leads to a gradual suppression of a target population [36]. *Wolbachia* represent a group of intracellular endosymbiotic bacteria which are considered the most ideal candidate for IIT. This bacterium can cause CI, male-killing, feminization of genetic males and parthenogenesis induction [37,38]. Among these, CI is the most common phenotype that *Wolbachia* impose on their hosts [39] and can be either unidirectional or bidirectional [40]. Unidirectional CI can occur when infected males from one strain mate with uninfected females from a different strain while the reciprocal cross is compatible; bidirectional CI can occur when both strains are infected with different *Wolbachia* and crosses in both directions are incompatible [40].

The ability of *Wolbachia* to cause CI along with its widespread nature has led to interest in its use as a potential environmentally-friendly biocontrol agent. Studies on IIT to control populations of insect disease vectors and agricultural pests have achieved encouraging results both in the field and laboratory. These include field experiments on *Aedes polynesiensis* in the South Pacific islands [41], on *Culex quinquefasciatus* in the south-western Indian Ocean islands [42], on *Aedes albopictus* in riverine islands in Guangzhou, China [43], and on *Ae. aegypti* in semi-rural village settings in Thailand [44]. They also include laboratory experiments targeting the medfly *Ceratitis capitata* [45]. Compared with other sterile insect techniques (SIT) which involve radiation or genetic modification of males, *Wolbachia*-based IIT seems to impose a relatively low fitness burden on released males and the method is not governed by challenging regulatory pathways [46].

Even though *Wolbachia* endosymbionts have not yet been used in the control of leaf-mining insects, they have been described from some leafminers including *L. trifolii* where they cause CI [47,48]. Apart from affecting the reproduction of hosts, *Wolbachia* may also have other important effects on the life history of leaf-mining herbivorous insects. For instance, in the phytophagous leaf-mining moth, *Phyllonorycter blancardella* (Lepidoptera: Gracillariidae) *Wolbachia* affects host plant physiology, producing the ‘green-island’ phenotype (photosynthetically active green patches) that enhances larval fitness [49]. In addition, *Wolbachia* can alter mtDNA haplotypes and potentially cause reproductive isolation in leafminers, as recently documented in *Phytomyza plantaginis* Goureau (Diptera: Agromyzidae) where two mtDNA haplotypes separate parthenogenic and bisexual populations both infected by *Wolbachia* [50].

Given the potential applications of endosymbionts in pest control, we set out to investigate the incidence of *Wolbachia* in 13 dipteran leaf-mining species and (by comparing the relatedness of *Wolbachia* strains against a host phylogeny) the occurrence of horizontal transmission in the *Liriomyza* group (c.f. *Drosophila* [51]). We then investigated *Wolbachia* in *Liriomyza brassicae* (Riley), a non-pest agromyzid from southern Australia, where we identified a similar *Wolbachia* as present in the three pest *Liriomyza* species. We were unable to have cultures of these three species as they are not present in southern Australia. In *L. brassicae*, we considered *Wolbachia* density across developmental stages and sexes, and the ability of *Wolbachia* to generate CI. This initial work represents a first step in a longer-term goal of exploring the feasibility of using *Wolbachia*-induced CI as a novel environmentally friendly tool for the control of *Liriomyza* pests.

## 2. Materials and Methods

### 2.1. Insect Materials, L. brassicae Cultures and Antibiotic Treatments

**Figure 1:**
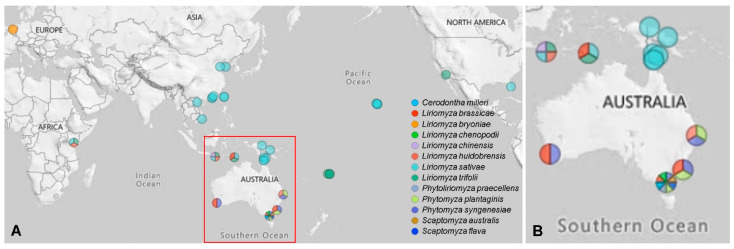
Sampling locations for leaf-mining species in this study. Sampling sites less than 200 km apart were merged, and the colors represent different leaf-mining species. (**A**) Overall sampling map; (**B**) Enlarged view of the red frame in (**A**).

Six agromyzid leaf-mining species (*Liriomyza brassicae*, *Liriomyza chenopodii*, *Phytomyza plantaginis*, *Phytomyza syngenesiae*, *Phytoliriomyza praecellens* and *Cerodontha milleri*) and two drosophilid leafminers (*Scaptomyza australis* and *Scaptomyza flava*) were collected from different locations in Australia. *Liriomyza brassicae* collections were also supplemented by overseas collections from collaborators. Specimens of *L. sativae*, *L. trifolii*, *L. huidobrensis*, *L. chinensis* and *L. bryoniae* were obtained from multiple locations and various hosts around the world (Figure 1, Table S1). Specimens were preserved in 95% ethanol and stored at −80 °C or for a few days at −20 °C until use. Species identifications were confirmed by Mallik Malipatil (Agriculture Victoria, AgriBio, La Trobe University, Bundoora, Australia) and verified by DNA barcoding based on mitochondrial COI [8].

A laboratory-reared strain of *Wolbachia*-infected *L. brassicae* was established from individuals collected from Flemington Bridge (latitude −37.787, longitude 144.939) in Melbourne, Victoria, Australia. The strain was reared on bok choy (*Brassica rapa* ssp. *chinensis*) at 25 °C, 60–70% relative humidity under a photoperiod of 16L:8D in 30 × 30 × 62 cm insect-proof cages. Bok choy was grown from seeds (Eden Seeds, Beechmont, Australia). Plants at 6–7 true leaf stage at one month old (Growth Stage 1–Leaf Production) were used for rearing flies. This population had been maintained in the laboratory for over a year (around 18 generations) at the time of the experiments.

To develop an uninfected *L. brassicae* strain, we placed a bunch of petioles of developed and healthy bok choy true leaves in 1 mg/mL tetracycline hydrochloride solution in a container (6.5 cm diameter base, 8 cm high) for two days to let leaves fully absorb the solution. The container was wrapped in foil to avoid tetracycline photodegradation. Meanwhile, 20 unmated infected pairs were placed in 35 mL vials (Genesee Scientific, San Diego, CA, USA) separately and covered with mesh, with honey streaked on the mesh as a food source. Two days later, leaves in good condition (i.e., turgid, unwilted) were provided to 20 pairs of flies separately in polypropylene cups (6.5 cm diameter base, 9 cm diameter top, 14 cm high). The lids of the cups were perforated and covered with mesh. Honey was streaked on the mesh to increase the longevity of mating pairs. We collected female and male individuals from the cups after three days to detect their *Wolbachia* infection status by using quantitative PCR (qPCR) (see below). Mined bok choy leaves were placed in fresh tetracycline solution until pupae were collected. When the next generation emerged, we selected a subset of flies to check their *Wolbachia* infection status, and the remaining flies were treated with tetracycline for two further generations following the methods described above. We then established 20 iso-female lines and generated lines from the offspring of parents which were completely cured of *Wolbachia* as assessed by qPCR (see below, no *Wolbachia* signal detected). We cultured these lines for another three generations to expand the *Wolbachia* cured colony.

### 2.2. Wolbachia Detection in 13 Dipteran Leaf-Mining Species

DNA was extracted from single individuals using the Chelex^®^ 100 resin (Bio-Rad, Hercules, CA, USA) method [8]. To confirm infection status, conventional PCR was performed, and the amplification of the *wsp* (*Wolbachia* surface protein) gene was taken as evidence of the presence of *Wolbachia*. The universal primer set was used to obtain *wsp* sequences, and the amplification was performed following an established protocol (http://pubmlst.org/wolbachia (accessed on 1 June 2021)) [52]. For the species *P. plantaginis* and *P. syngenesiae,* new *Wolbachia*-specific primers were designed to increase the specificity of *wsp* sequences given that universal primers were not specific enough (Table S2). The PCR thermal conditions were the same as above. PCR products were sent to Macrogen (Seoul, Korea) for purification and Sanger sequencing. *Wolbachia* infected leafminer specimens verified by sequencing were always included as positive controls, and water was used as a negative control in all *Wolbachia* screening.

### 2.3. MLST System for Wolbachia Classification

*Wolbachia* supergroup designations are routinely used to describe the major phylogenetic subdivisions of this bacterial group [52]. Phylogenetic analyses based on *wsp* remain the primary methods for *Wolbachia* supergroup designations [53]. However, due to the level of recombination in *Wolbachia pipientis* and close relatives, reliable *Wolbachia* strain characterization requires a multi-locus strain typing (MLST) approach [52,54]. Here, we selected two agromyzid species (*P. praecellens* and *L. brassicae*) and used a standard multi-locus strain typing (MLST) approach to validate their supergroup status as determined from the *wsp* gene. We identified the *Wolbachia* strains by comparing results with the MLST database. The MLST sequences of these two species were concatenated into a supergene alignment with 2079 nucleotides based on five housekeeping genes (gatB, *coxA*, *hcpA*, *ftsZ* and *fbpA*), which were amplified with universal primers and the methods followed the published protocol (http://pubmlst.org/wolbachia (accessed on 1 June 2021)) [52]. PCR products were sequenced directly as described earlier.

### 2.4. Wolbachia Density Estimation in L. brassicae

For rapid monitoring of *Wolbachia* in *L. brassicae*, we developed a robust screening assay that was able to simultaneously detect *Wolbachia* infection and quantify density in *L. brassicae*. We selected *actin* as a housekeeping gene due to its stability across developmental stages and sexes [55]. The sequences of the *L. sativae actin* gene [56] (GenBank No. DQ452369) and *L. brassicae wsp* gene from this study (GenBank No. MW047082) were used to design specific qPCR primers through an online primer designing tool (https://www.ncbi.nlm.nih.gov/tools/primer-blast/ (accessed on 1 June 2021)). A standard curve and sensitivity analyses were applied to assess the performance of the qPCR assay and estimate its efficiency (Figures S1 and S2).

*Wolbachia* density of *L. brassicae* in different developmental stages and different generations were quantified using qPCR. Genomic DNA was extracted from F0, F1 and F2 generations of *L. brassicae,* including the 3rd instar larvae (24 individuals), 5-day-old pupae (24 individuals) and adult flies emerged within 24 h (12 females and 12 males). DNA was extracted as described above and then diluted 1: 2 with DEPC treated water. 2 μL of this diluted DNA was used as a template in real-time quantitative PCR using *actin* and *wsp* primers (Table S2). PCR reactions were performed using a Roche LightCycler^®^ 480 system following the cycling conditions outlined by Lee and colleagues [57], except that the annealing temperature was 55 °C for 20 s instead of 58 °C for 15 s (the optimal annealing temperature was determined by conventional gradient PCR in this study). Each sample had three technical replicates, and Cp average values were applied for analyses. Differences between the Cp of the *wsp* and *actin* of *L. brassicae* individuals were transformed by 2^[(Cp of *actin*) − (Cp of *wsp*)]^ to obtain approximate estimates of *Wolbachia* density.

### 2.5. Crossing Experiments

Individual puparia collected from infected lines and treated lines were put in separate 1.7 mL centrifuge tubes to emerge. In different crossing conditions, crosses were established with a single male and a single female. Sexes of adults were identified under a dissecting microscope, and crosses were performed within 24 h of adult emergence. For each cross, a pair of virgin individuals were put in 35 mL vials (Genesee Scientific, San Diego, CA, USA). Vials were covered with mesh and honey was streaked onto the mesh to prolong the lifespan of flies. To increase the chance of mating (which normally occurs 10–12 h after emergence [58]), pairs were left for 48 h and then transferred to insect-proof cages (20 cm × 17.5 cm × 12.5 cm) with two developed and healthy bok choy true leaves (one-month-old) placed in a 5.5 cm diameter base × 4 cm diameter top × 10 cm high conical bottle containing water and sealed with parafilm to avoid flies drowning in the water. The lids of cages were cut out and partly covered with mesh so honey could spread on the surface. Three days later, live pairs of flies were placed in 100% ethanol for subsequent validation of the *Wolbachia* status. The mined leaves were kept in 150 mL polypropylene boxes (FPA Australia Pty Ltd., Melbourne, Australia) lined with paper towel and monitored every day. The number of 1st instar larvae (determined under the microscope), puparia and adults of each sex were counted. Each cross was replicated six times.

### 2.6. Data Analyses

The *wsp* sequences were aligned and edited with Geneious 9.1.8 [59]. For phylogenetic analysis, sequences of the *wsp* gene and MLST genes from a range of species were retrieved from GenBank and analyzed together with our data. The MLST sequences were concatenated into a supergene alignment with 2079 nucleotides. Nucleotide diversity was calculated using DnaSP 6 [60]. The haplotypes detected in the present study were compared with published sequences. Identical sequences were removed from the final data set so that each haplotype was only represented once. The relationship of leafminer species and their corresponding *Wolbachia* infection was analyzed with maximum likelihood (ML) inference using IQtree 1.4.2 [61]. To assess nodal support, we performed 1000 ultrafast bootstrap replicates and a SH-aLRT test with 1000 replicates. A MLST phylogenetic tree was generated using the Neighbour-Joining (NJ) method [62] through MEGA X [63] based on Kimura 2-parameter distances with 1000 replicates of bootstrapping [64]. DNA sequences from the present study have been submitted to GenBank.

Statistical analyses of experimental data were performed using SPSS statistics version 24.0 for Windows (SPSS Inc., Chicago, IL, USA). *Wolbachia* densities in the larval and pupal stages of *L. brassicae* were analyzed using general linear models (GLMs), with life stage and generation included as factors. We performed a separate analysis for *Wolbachia* density in adults, with sex and generation included as factors. Pairwise comparisons between life stages within a generation were undertaken with t tests. For crossing experiments of *L. brassicae*, the normal distribution of data was checked with a Kolmogorov-Smirnov test in Graphpad Prism (GraphPad Software Inc., San Diego, CA, USA). We then used one-way ANOVA tests to compare offspring numbers and sex ratios (after arcsin transformation) between crosses, excluding the cross between *Wolbachia*-infected males and uninfected females which produced no offspring. We also ran pairwise comparisons (*t*-tests) to test the effect of tetracycline treatment on the offspring number and sex ratio of offspring from infected and uninfected female parents (when mated with uninfected males) and offspring from infected and uninfected male parents (when mated with uninfected females) to assess any potential effects of antibiotic treatments.

## 3. Results

### 3.1. Wolbachia Detection Based on wsp Sequences This Population Had Been Maintained

Leaf-mining species from different populations were tested for *Wolbachia* infection status through the amplification of a fragment of the *wsp* gene. We found that all individuals of *L. huidobrensis*, *L. chinensis*, *L. bryoniae*, *L. brassicae, L. chenopodii, P. plantaginis, P. syngenesiae, P. praecellens, S. australis and S. flava* tested were positive for *Wolbachia*, while all *C. milleri* individuals tested negative. For *L. trifolii*, individuals from four countries (USA (a laboratory colony), Kenya, Timor-Leste and Fiji) were positive for *Wolbachia*, but those from Indonesia (a recent incursion) were negative. For *L. sativae, Wolbachia* frequencies differed between populations (Table 1 and Table S1). Of the 27 populations tested for this species, four populations (Sao Bay-Vietnam, Vero Beach-USA (a laboratory colony), Liquisa–Timor-Leste and Ermera-Timor-Leste) showed a high proportion of individuals (>90%) positive for *Wolbachia*. An additional three populations (Thursday Island–Australia, Seloi Kraik-Timor-Leste and Seloi Malere-Timor-Leste) had only a single individual positive for *Wolbachia*.

**Figure 2:**
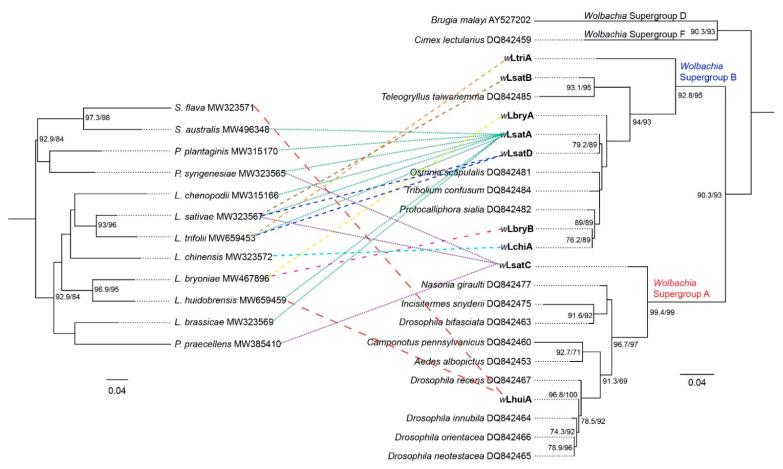
Maximum-likelihood phylogenetic tree to reveal relationships among *Wolbachia* strains and leafminer species. The phylogeny is inferred by IQTREE based on 3′ end COI sequences of leaf-mining species, and *wsp* sequences of *Wolbachia* detected from corresponding leaf-mining species. Published *wsp* sequences from other insect species were included to allocate the infections to *Wolbachia* supergroups (the *Wolbachia wsp* alleles in this study are labeled in bold, additional *wsp* alleles refer to Baldo et al. [52]). Numbers beside nodes are IQTREE ultrafast bootstrap and SH-aLRT values. ML bootstrap values > 60% are shown on branches.

We constructed a maximum likelihood (ML) tree to link different leaf-mining species and their *Wolbachia* by using the 3′ region of COI for species and partial sequences of the *wsp* gene. Prior published *wsp* sequences from other insect species were included to allocate the new infections to *Wolbachia* supergroups [52] (Figure 2). The sequence alignment based on the *wsp* sequences suggests the same leaf-mining species can be infected with different *Wolbachia* strains (Figure 2, Table 2 and Table S3). We found four different *wsp* alleles (*w*LsatA, *w*LsatB, *w*LsatC and *w*LsatD) in *L. sativae* from different populations, which span phylogenetic supergroups A and B. The *wsp* alleles of *L. sativae* from Sao Bay (Vietnam) was *w*LsatC which belongs to *Wolbachia* subgroup A. On the other hand, *wsp* alleles of *L. sativae* from Timor-Leste (containing two *Wolbachia* alleles: *w*LsatA and *w*LsatB) and Thursday Island (*w*LsatD) belonged to *Wolbachia* subgroup B. Additionally, the alignment of *wsp* sequences showed that *L. trifolii* detected in Kirinyaga (Kenya) was *w*LtriA, which was different to *L. trifolii* USA, Fiji and Japan (*w*LsatD) and Timor-Leste (*w*LsatA). Furthermore, we found the *wsp* alleles of *L. huidobrensis* from Bali (Indonesia) and Nairobi County (Kenya) were *w*LhuiA, whereas the *wsp* allele detected in *L. huidobrensis* from Tarome and Kalabar (Australia) was *w*LsatA.

At the same time, our results suggest that different leaf-mining species may share the same *Wolbachia* strain (Figure 2, Table 2 and Table S3). The sequence alignment results based on *wsp* indicated that *Wolbachia* alleles from *P. praecellens* from Royal Park (Australia) and *L. sativae* from Sao Bay (Vietnam) were both *w*LsatC. *w*LsatA was the most common allele, shared by eight leaf-mining species (*L. huidobrensis*, *L. sativae, L. trifolii*, *L. brassicae, L. chenopodii, P. plantaginis, P. syngenesiae* and *S. australis*). We also found that the *Wolbachia* allele in *L. trifolii* (California-USA, and Fiji) and in *L. sativae* (Thursday Island-Australia) were identical to the sequence from *L. trifolii* mentioned in Tagami et al. [47] when comparing their *wsp* sequences (*w*LsatD). It is worth noting that there is only a single base-pair difference between *w*LsatA and *w*LsatD. Moreover, the *wsp* allele of *L. huidobrensis* (Indonesia and Kenya) and *S. flava* (Australia and Hawaii) were both *w*LhuiA, which suggests that *L. huidobrensis* and *S. flava* may share the same *Wolbachia* strain. Interestingly, *Wolbachia* superinfections were detected in *P. syngenesiae* in one population (Flemington Bridge–Australia) based on sequencing data; we found 192 individuals were infected with *w*LsatA as determined from screens with primers specific to this infection, but three of these individuals were diagnosed as having two different *wsp* alleles (*w*LsatA and *w*LsatC) based on universal primers. In eight other Australian populations, only one allele (*w*LsatA) was found in *P. syngenesiae* (Table 2 and Table S3).

### 3.2. MLST System for Wolbachia Classification

**Figure 3:**
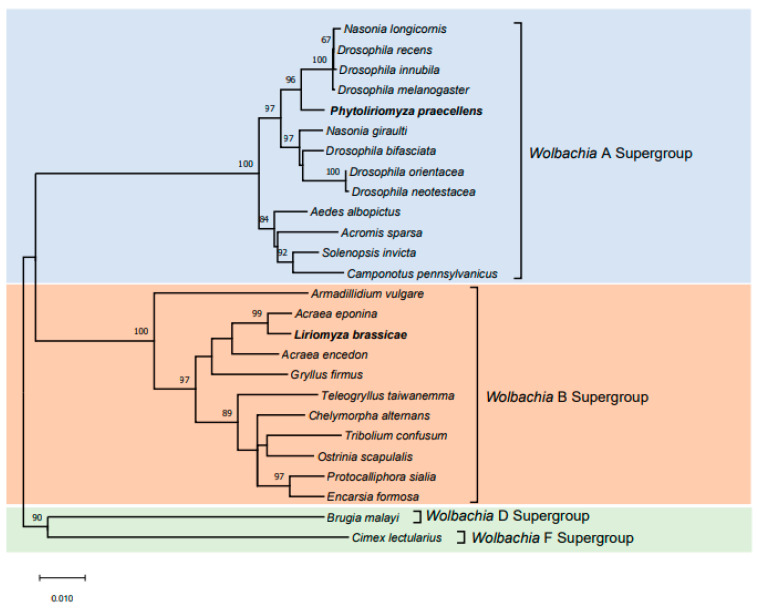
Phylogenetic placement of *Wolbachia* from two leaf-mining species (*Phytoliriomyza praecellens* and *Liriomyza brassicae*) collected in Australia based on MLST genes within a collection of insects taken from Baldo et al. [52]. The neighbor-joining tree (K2P model, 1000 bootstrap replicates) is based on multiple alignments of concatenated DNA sequences encoding the *coxA*, *fbpB*, *ftsZ*, *gatB* and *hcpA* genes. Bootstrap values are shown for all nodes. ML bootstrap values > 60% are shown on branches. The two species included in this study are shown in bold.

Our study demonstrates that the MLST phylogenetic analyses were consistent with the above *wsp* phylogenetic analyses; the *Wolbachia* from *P. praecellens* was allocated to the A-supergroup, and the infection from *L. brassicae* was allocated to the B-supergroup (Figure 3). Allelic profiles for the five housekeeping genes of *Wolbachia* from *P. praecellens* were (22) *gatB*, (23) *coxA*, (24) *hcpA*, (3) *ftsZ* and (23) *fbpA*. A comparison of sequences from the five MLST genes with those in the databases was consistent with the notion that the *Wolbachia* strain detected in *P. praecellens* represents *Dsim_A_wRi*, which was also identified in *Drosophila simulans* and shows CI [52,65]. For *L. brassicae*, the five allelic profiles were (9) *gatB*, (280) *coxA*, (40) *hcpA*, (7) *ftsZ* and (9) *fbpA*. A comparison of sequences from the five MLST genes with those in the databases suggested that this *Wolbachia* strain represented a new strain, which we have named *Lbra_B_1*.

### 3.3. Wolbachia Density Estimation in L. brassicae

**Figure 4:**
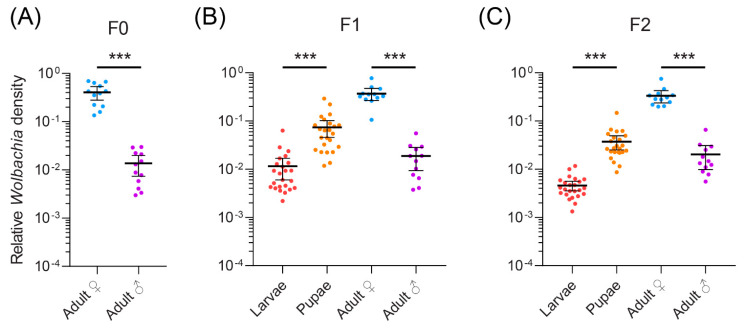
*Wolbachia* density in (**A**) F0, (**B**) F1 and (**C**) F2 generations across different developmental stages and sexes of *Liriomyza brassicae*. Dots represent data from individual flies while horizontal lines and error bars are means and 95% confidence intervals. Data are shown on a log scale. Significance levels for comparisons between sexes and life stages within a generation are shown (*t*-tests, ***, *p* < 0.001).

Given that *Wolbachia* strain *Lbra_B_1* is prevalent among different leaf-mining species, we selected *L. brassicae* as a model species to investigate its *Wolbachia* density across different generations and developmental life stages. *Wolbachia* density was quantified using qPCR. In adults, *Wolbachia* density was stable across three generations, with no significant effect of generation on density (General Linear Model: F_2,66_ = 0.194, *p* = 0.824). However, *Wolbachia* density differed between the sexes, with females having a higher density than males (F_1,66_ = 416.216, *p* < 0.001) (Figure 4). This difference was evident in each generation (Figure 4). *Wolbachia* density increased during development, with higher densities in puparia compared with larvae (F_1,92_ = 186.528, *p* < 0.001), a difference that was evident in both the F1 and F2 generations (Figure 4) There was also a significant effect of generation in this comparison, with higher densities in the F1 generation compared with the F2 generation (F_1,92_ = 18.274, *p* < 0.001).

### 3.4. Crossing Experiment of L. brassicae

To determine the influence of *Wolbachia* infection on *L. brassicae*, we performed reciprocal crosses between *Wolbachia*-infected and treated individuals. High hatching rates were observed when the crosses were performed between the same strains or between infected females and treated males (Table 3). The number of larvae/puparia/adults did not differ between crosses when the incompatible cross was excluded (One-way ANOVA: larvae: F_2,15_ = 0.413, *p* = 0.669; puparia: F_2,15_ = 0.566, *p* = 0.580; Adults: F_2,15_ = 0.448, *p* = 0.648). The emergence rate of adults in these crosses was also relatively high, while sex ratios did not differ significantly from 1:1 in any cross where flies emerged (t-test, theoretical mean of 50% males or females, all *p* > 0.05). However, no leafmines were observed in crosses between infected males and treated females, and no offspring emerged, indicating complete CI (Table 3).

## 4. Discussion

In recent years, there has been increasing interest in the biology of *Wolbachia* and its application as a tool for the management or modification of insect populations. The distribution of *Wolbachia* among its dipteran leafminer hosts represents an important first step in developing such applications. This study presents the first broad-scale study to screen natural populations of leaf-mining flies for *Wolbachia*, mainly focusing on the Agromyzidae from Australia. Our results indicate that *Wolbachia* is present in 10 agromyzid species (*L. sativae*, *L. huidobrensis*, *L. trifolii*, *L. bryoniae*, *L. chinensis*, *L. brassicae*, *L. chenopodii*, *P. plantaginis*, *P. syngenesiae* and *P. praecellens*) and two drosophilid species (*S. flava* and *S. australis*).

While the Australian specimens we considered were largely adventive species, the specimens of *P. praecellens*, *C. milleri*, *L. chenopodii* and *S. australis* represent indigenous species. Both groups clearly show a potentially high incidence of *Wolbachia* infection. More extensive sampling of these species is required to establish the incidence of *Wolbachia* across the geographic range of these species, since *Wolbachia* infection frequencies in leafminers and other insects can be quite variable. In Japan, Tagami et al. [48] previously found that 40 *L. trifolii* individuals were infected out of 226 tested. They detected *Wolbachia* in only one out of eight field collections and found infections present at different frequencies within laboratory colonies, consistent with our results showing a variable incidence of *Wolbachia* in this species. Tagami et al. [48] also found that five collections of *L. sativae* (116 individuals) were uninfected, which contrasts with the fact that we found several infected *L. sativae* populations. Overall, our field collections suggest that the incidence of *Wolbachia* across leafminers species and within species is high, with only *C. milleri* uninfected by *Wolbachia,* but the *L. sativae* and *L. trifolii* results also highlight the potentially high level of polymorphism present in some species.

The phylogenetic tree based on *wsp* sequences obtained from all agromyzid and drosophilid species analysed indicates seven *Wolbachia* strains (*w*LsatA, *w*LsatB, *w*LsatD, *w*LbryA, *w*LbryB, *w*LchiA and *w*LtriA) belonging to *Wolbachia* subgroup B and two *Wolbachia* strains (*w*LhuiA and *w*LsatC) belonging to *Wolbachia* subgroup A. Tagami et al. [47,48] detected two strains of *Wolbachia* in *L. trifolii* and *L. bryoniae* (both from *Wolbachia* B supergroups) and showed that one strain of *Wolbachia* (*w*LsatD) induced strong CI in *L. trifolii*. In our study, *w*LsatA was the most common strain shared by eight different leaf-mining flies and only one base-pair different from *w*LsatD based on *wsp*. MLST analysis indicates that the *w*LsatA based *Wolbachia* strain of *L. brassicae* is a new *Wolbachia* strain (*Lbra_B_1*), and the crossing experiments indicate that this strain causes strong CI effect on hosts. Additionally, we note that the sequence of the *wsp* allele *w*LsatC (found in *P. praecellens* and *P. syngenesiae*) is the same as the *Wolbachia w*Ri strain. This *Wolbachia* strain was detected in *Drosophila simulans* in the 1980s in southern California and has been shown to induce strong CI [65].

The presence of *Wolbachia* in the vast majority of leafminers surveyed here and the fact it is near fixation in many species, suggests that *Wolbachia* spreads relatively easily in this group through horizontal transmission. Natural *Wolbachia* infections can spread from low initial frequencies [51]. “Super spreader’’ *Wolbachia*, aided by reproductive manipulations such as CI, tend to spread readily due to low fitness costs in novel hosts and high maternal transmission rates [51]. In this study, the superinfection (*w*LsatA and *w*LsatC) we detected at one site in *P. syngenesiae* based on the *wsp* sequencing suggests that phylogenetically distant *Wolbachia* strains may be readily maintained in the same leaf-mining species. This suggests that at least some species of agromyzids would be ready recipients of *Wolbachia* infections, increasing the likely success of microinjections. A high rate of natural horizontal transfer of *Wolbachia* in leafminers may account for the presence of similar *Wolbachia* strains in unrelated species. Plant-mediated horizontal transmission might explain the high *Wolbachia* infectious rate within phytophagous leaf-mining species and the presence of identical strains in evolutionarily distant species [66]. All of these findings point to the presence of *Wolbachia* strains with strong CI and high rates of horizontal transmission in leafminers suitable for future manipulations.

For *L. sativae*, prior studies have suggested that all members of the *L. sativae*-A clade (22 specimens tested) were infected with a single *Wolbachia* subgroup A strain [4], whereas none of the members of the *L. sativae*-W+L clades (86 specimens tested) had this infection. Parish et al. [67] found no *Wolbachia* infections in *L. sativae* (Clade-B) from Brazil (64 specimens tested). However, our study indicates that some populations of the *L. sativae*-W clade are infected by the *Wolbachia* subgroup A strain, while others are infected by the subgroup B strain. Moreover, we found that *L. sativae* (haplotype S.01, Clade-A) from Florida was infected with *Wolbachia* (NCBI blast tool showed ~93% similar to *Wolbachia* endosymbiont of *Aptinothrips stylifer*, GenBank: MT224213) although we excluded this sample due to poor sequence quality. Our *Wolbachia* screening results are therefore consistent with Scheffer and Lewis [4] in indicating that *L. sativae*-A clade is infected with *Wolbachia*. Detection of *Wolbachia* in other clades may depend on geographical context and perhaps sample size. Parish et al. [67] did not find *Wolbachia* infections in *L. brassicae* (12 specimens) (note the CO1 haplotypes of *L. brassicae* in Brazil are B.02-B.05, while the haplotype from Australia and Timor-Leste is B.01), *L. huidobrensis* (6 specimens) and *Calycomyza malvae* (6 specimens). These results contrast with our findings on larger samples of *L. brassicae* and *L. huidobrensis*, with all populations of these species that were tested having *Wolbachia*. Some populations of *L. sativae, L. trifolii* and *L. huidobrensis* shared the same *wsp* allele *w*LsatA, and it is possible that populations of these species are infected with *Wolbachia* strain *Lbra_B_1* and cause CI.

Selection on *Wolbachia* tends to operate via the female host to favor strong maternal transmission to the female generation, while a low level of *Wolbachia* in males could reduce any deleterious fitness effects associated with the *Wolbachia* infection [68]. Consistent with this prediction and the pattern seen in some *Drosophila* [68], we found that *Wolbachia* in *L. brassicae* differed substantially between the sexes and this may be a consequence of sex-specific selection associated with transmission. It remains to be seen if this sex difference in density is also present in other *Liriomyza* species. It would be interesting to compare the competitiveness of male flies with natural *Wolbachia* infections against those sterilized through irradiation.

Following the incursion of *L. sativae* and *L. huidobrensis* into Australia, there have been recent detections of *L. trifolii* in the Torres Strait (QLD, Australia), in Kununurra (WA, Australia) and in the Northern Peninsula Area of Cape York Peninsula in 2021 [10]. It will be worth tracking the *Wolbachia* status of all these incursions in a range of locations, and to establish patterns of CI across infections from these species to test for the feasibility of an IIT strategy. Tagami et al. [47] raised several challenges that would need to be overcome for an IIT approach against leafminers. In particular, IIT may be too expensive if flies are raised on plants rather than on artificial media, and an efficient sex sorting system would need to be developed. Sultan et al. [69] looked at mechanical sorting of puparia of *L. trifolii* but this proved difficult, and a genetic sexing method may be required. IIT would likely be restricted to greenhouses initially given costs. IIT may also be more feasible if used against small populations early in the growing season, and if IIT is combined with parasitoids such as *Dacnusa sibirica,* which is often released early in the growing season overseas to supplement overwintering *D. sibirica* [70]. In these systems, *D. isaea* is then released weekly once leafminer numbers build up later in the season [71]. As apparent from experience with SIT, an IIT method will likely need to be combined with other IPM strategies such as parasitoid releases, which represent an effective way of suppressing *Liriomyza* pests [35,71]. Additionally, the combination of an IIT approach with traditional insecticides seems cost-effective and promising, where insecticides are used to reduce the population to a lower level before using an IIT approach.

Overall, results from this study provide information for understanding *Wolbachia* infections in different leaf-mining species, as well as patterns of variation within species. Our findings indicate a high incidence of infection within and across species and suggest that transfections across species are likely to have a high chance of success when using other *Liriomyza* or dipteran species as infection sources. Our results provide a starting point for developing *Wolbachia* as a future biocontrol agent. Future work could consider further genomic analysis of the *Wolbachia* strains from different leafminers species, tissue localization of the *Wolbachia* infection and the generation of transinfections to investigate incompatibility relationships among the *Wolbachia* from the different leafminers species.

## Figures and Tables

**Table 1:** Overall *Wolbachia* infection rates in leafminer species at the population and individual levels.

Species	Populations Tested	Populations Positive for *Wolbachia*	Individuals Tested	Individuals Positive for *Wolbachia*
*Liriomyza sativae*	27	7/27	328	44/328
*Liriomyza trifolii*	7	6/7	63	51/63
*Liriomyza huidobrensis*	4	4/4	45	45/45
*Liriomyza bryoniae*	1	1/1	20	20/20
*Liriomyza chinensis*	1	1/1	12	12/12
*Liriomyza brassicae*	11	11/11	342	342/342
*Liriomyza chenopodii* *	5	5/5	120	120/120
*Phytomyza plantaginis*	7	7/7	173	173/173
*Phytomyza syngenesiae*	9	9/9	360	360/360
*Phytoliriomyza praecellens* *	1	1/1	8	8/8
*Cerodontha milleri* *	2	0/2	24	0/24
*Scaptomyza australis* *	3	3/3	72	72/72
*Scaptomyza flava*	2	2/2	12	12/12

* Species native to Australia.

**Table 2:** *Wolbachia* infection supergroup allocation and allele names corresponding to host species based on *Wolbachia wsp* sequences.

Species	Country	Location	*Wolbachia* Supergroup	*Wolbachia wsp* Allele
*Liriomyza sativae*	Timor-Leste	Seloi Malere, Aileu	B	*w*LsatA
*Liriomyza sativae*	Timor-Leste	Seloi Kraik, Alieu	B	*w*LsatB
*Liriomyza sativae*	Timor-Leste	Liquisa, Dato	B	*w*LsatA
*Liriomyza sativae*	Timor-Leste	Ermera, Mertutu	B	*w*LsatA
*Liriomyza sativae*	Vietnam	Sao Bay	A	*w*LsatC
*Liriomyza sativae*	Australia	Thursday Island, QLD	B	*w*LsatD
*Liriomyza trifolii*	USA	California	B	*w*LsatD
*Liriomyza trifolii*	Kenya	Kirinyaga County	B	*w*LtriA
*Liriomyza trifolii*	Japan	Shizuoka, Hamamatsu	B	*w*LsatD
*Liriomyza trifolii*	Timor-Leste	Bazartete, Leoreka	B	*w*LsatA
*Liriomyza trifolii*	Japan	Miyagi	B	*w*LsatD
*Liriomyza trifolii*	Fiji	Qereqere, Sigatoka Valley	B	*w*LsatD
*Liriomyza trifolii*	Fiji	Wainibokasi, Nausori	B	*w*LsatD
*Liriomyza trifolii*	Fiji	Koronivia, Nausori	B	*w*LsatD
*Liriomyza huidobrensis*	Indonesia	Bali	A	*w*LhuiA
*Liriomyza huidobrensis*	Kenya	Nairobi County	A	*w*LhuiA
*Liriomyza huidobrensis*	Australia	Tarome, QLD	B	*w*LsatA
*Liriomyza huidobrensis*	Australia	Kalabar, QLD	B	*w*LsatA
*Liriomyza bryoniae*	Netherlands	Berkel en Rodenrijs	B	*w*LbryA
*Liriomyza bryoniae*	Japan	Hamamatsu, Shizuoka	B	*w*LbryB
*Liriomyza chinensis*	Indonesia	Tabanan Regency, Bali	B	*w*LchiA
*Liriomyza brassicae*	Timor-Leste	Seloi Malere	B	*w*LsatA
*Liriomyza brassicae*	Timor-Leste	Seloi Kraik	B	*w*LsatA
*Liriomyza brassicae*	Australia	Melbourne locations, VIC ^a^	B	*w*LsatA
*Liriomyza brassicae*	Australia	Bruce, ACT	B	*w*LsatA
*Liriomyza brassicae*	Australia	Lesmurdie, WA	B	*w*LsatA
*Liriomyza chenopodii*	Australia	Melbourne locations, VIC ^b^	B	*w*LsatA
*Phytomyza plantaginis*	Australia	Flemington Bridge, VIC	B	*w*LsatA
*Phytomyza plantaginis*	Australia	Glenrowan, VIC	B	*w*LsatA
*Phytomyza plantaginis*	Australia	Stanhope, VIC	B	*w*LsatA
*Phytomyza plantaginis*	Australia	Elmore, VIC	B	*w*LsatA
*Phytomyza plantaginis*	Australia	Romsey, VIC	B	*w*LsatA
*Phytomyza plantaginis*	Australia	Lismore, NSW	B	*w*LsatA
*Phytomyza plantaginis*	Australia	Bruce, ACT	B	*w*LsatA
*Phytomyza syngenesiae*	Australia	Flemington Bridge, VIC	A/B	*w*LsatC/*w*LsatA
*Phytomyza syngenesiae*	Australia	Werribee South, VIC	B	*w*LsatA
*Phytomyza syngenesiae*	Australia	Glen Waverley, VIC	B	*w*LsatA
*Phytomyza syngenesiae*	Australia	Fitzroy North, VIC	B	*w*LsatA
*Phytomyza syngenesiae*	Australia	Werribee VIC	B	*w*LsatA
*Phytomyza syngenesiae*	Australia	Bruce, ACT	B	*w*LsatA
*Phytomyza syngenesiae*	Australia	Yanakie, VIC	B	*w*LsatA
*Phytomyza syngenesiae*	Australia	Lesmurdie, WA	B	*w*LsatA
*Phytomyza syngenesiae*	Australia	Ballina, NSW	B	*w*LsatA
*Phytoliriomyza praecellens*	Australia	Royal Park, VIC	A	*w*LsatC
*Scaptomyza flava*	Australia	Flemington Bridge, VIC	A	*w*LhuiA
*Scaptomyza flava*	Australia	Shoreham, VIC	A	*w*LhuiA
*Scaptomyza australis*	Australia	Royal Park, VIC	B	*w*LsatA
*Scaptomyza australis*	Australia	Flemington Bridge, VIC	B	*w*LsatA
*Scaptomyza australis*	Australia	Shoreham, VIC	B	*w*LsatA

^a^ Includes Flemington Bridge, Gladstone Park, Northcote, Fitzroy North, Thomastown, Werribee and Werribee South populations. ^b^ Includes Flemington Bridge, Werribee, Werribee South, Glen Waverley, Fitzroy North and Werribee populations. QLD = Queensland, VIC—Victoria, ACT = Australian Capital Territory, WA = Western Australia, NSW = New South Wales.

**Table 3:** Crossing experiments to detect the influence of *Wolbachia* infection on *Liriomyza brassica* offspring. The number of 1st instar, pupae and adults are shown, along with the percent emergence of puparia and the percent of offspring that were female. The difference between offspring from uninfected and infected females (when mated to uninfected males) and the difference between offspring from uninfected males and infected males (when mated with infected females) was also assessed via *t*-tests (on arcsin proportions in the case of proportional data) and are included below.

Cross	Female	Male	1st Instar ^a^	Pupae ^a^	Adults ^a^	Percent Emergence of Puparia	Percent of Female
1	Uninfected ^b^	Uninfected ^b^	148.1 ± 29.3	136.1 ± 29.1	114.5 ± 27.5	83.8 ± 5.7	56.6 ± 7.6
2		Infected	0	0	0	0	-
3	Infected	Uninfected ^b^	138.8 ± 26.9	125.6 ± 24.2	103.0 ± 18.3	82.4 ± 6.4	46.1 ± 3.9
4		Infected	133.5 ± 28.4	119.6 ± 27.9	102.8 ± 26.5	85.8 ± 6.1	54.1 ± 4.6
Statistical comparisons (*t*-test statistic, df, *p* value)							
1 versus 3		0.573, 10, 0.579	0.678, 10, 0.512	0.851, 10, 0.414	0.399, 10, 0.698	2.973, 10, 0.014	-
3 versus 4		0.333, 10, 0.745	0.396, 10, 0.699	0.012, 10, 0.990	0.920, 10, 0.378	3.216, 10, 0.009	-

^a^ Mean ± Standard Deviation; ^b^ Antibiotic-treated uninfected strain.

## Data Availability

The data presented in this study are available on request from the corresponding author.

## Supplementary Materials

The following are available online at https://www.mdpi.com/article/10.3390/insects12090788/s1, Table S1: Information on leaf-mining fly specimens collected in this study and their *Wolbachia* infection status. “Number +/− for *Wolbachia*” indicates fly numbers that were positive/negative to *Wolbachia*; Figure S1: Estimation of qPCR amplification efficiencies of *actin* and *wsp* of *Liriomyza brassicae*. The original DNA concentration was 1 ng/μL. Standard curves of qPCR used threefold serial dilutions (10 µL of dilution added to 20 µL of H_2_O) of *L. brassicae* DNA on three separate occasions under identical conditions; Figure S2: Agarose electrophoresis gel of conventional PCR (*actin* and *wsp*) of *Liriomyza brassicae*. Lanes 1–8: DNA diluted from 3^−1^ to 3^−8^ (threefold serial dilutions), Lane 9: negative control (water); Table S2: Specific primers used for quantitative PCR and DNA barcoding of the leafminer species and *Wolbachia*. For other species, universal primers from Baldo et al. [1] were used; Table S3: Taxonomic information of leaf-mining flies and *Wolbachia wsp* sequences. Allele names correspond to the taxonomic placement of the host species.
