# Supplementary material for: High Incidence of Related Wolbachia across Unrelated Leaf-Mining Diptera
Source: Insects. 2021 Sep 3;12(9):788. doi: 10.3390/insects12090788 (PMC8465256; doi:10.3390/insects12090788)
Supplement: Supplementary file 1 [file insects-12-00788-s001.zip › insects-1350454-supplementary.pdf]

## SUPPLEMENTARY MATERIALS

**Table S1.** Information on leaf-mining fly specimens collected in this study and their *Wolbachia* infection status. "Number +/- for *Wolbachia*" indicates fly numbers that were positive/negative to *Wolbachia.*

| Species | Collection date | Location | Latitude | Longitude | Host plant | Individuals tested | Life stage or sex | Number +/- for *Wolbachia* |
|---|---|---|---|---|---|---|---|---|
| *Liriomyza sativae* | 2017.07 | Seisia, QLD, Australia | -10.850 | 142.368 | *Macroptilium atropurpureum* | 20 | pupae | 0/20 |
| *Liriomyza sativae* | 2018.07 | Thursday Island, QLD, Australia | -10.575 | 142.221 | *Macroptilium atropurpureum* | 43 | pupae | 1/42 |
| *Liriomyza sativae* | 2019.06 | Masig Island, QLD, Australia | -9.751 | 143.408 | *Ricinus communis* | 6 | 3♀+3♂ | 0/6 |
| *Liriomyza sativae* | 2019.06 | Boigu Island, QLD, Australia | -9.274 | 142.223 | *Macroptilium atropurpureum* | 10 | larvae | 0/10 |
| *Liriomyza sativae* | 2019.11 | Vanimo, Sandaun, Papua New Guinea | -2.685 | 141.304 | *Cucumis sativus* | 13 | larvae | 0/13 |
| *Liriomyza sativae* | 2017.11 | Wewak, East Sepik Province, Papua New Guinea | -5.225 | 145.793 | *Solanum lycopersicum* | 22 | larvae | 0/22 |
| *Liriomyza sativae* | 2018.11 | Seloi Malere, Aileu, Timor-Leste | -8.704 | 125.609 | *Cucurbita pepo* | 9 | 3♀+6♂ | 1/8 |
| *Liriomyza sativae* | 2017.09 | Seloi Kraik, Aileu, Timor-Leste | -8.698 | 125.534 | *Cucurbita pepo* | 32 | 26♀+6♂ | 1/31 |
| *Liriomyza sativae* | 2017.09 | Liquisa, Dato, Timor-Leste | -8.604 | 125.293 | *Cucurbita* sp. | 1 | larvae | 1/0 |
| *Liriomyza sativae* | 2017.09 | Ermera, Mertutu, Timor-Leste | -8.757 | 125.400 | *Solanum lycopersicum* | 4 | larvae | 4/0 |
| *Liriomyza sativae* | 2019.04 | Bali, Indonesia | -8.340 | 115.091 | *Cucumis sativus* | 20 | 10♀+10♂ | 0/20 |
| *Liriomyza sativae* | 2020.01 | Lien Kiep, Vietnam | 11.739 | 108.360 | *Benincasa hispida* | 14 | larvae | 0/14 |

| | | | | | | | | |
|---|---|---|---|---|---|---|---|---|
| *Liriomyza sativae* | 2020.01 | Sao Bay, Vietnam | 20.592 | 105.601 | *Cucumis sativus* | 17 | larvae | 16/1 |
| *Liriomyza sativae* | 2016.07 | Shenzhen, Guangdong, China | 22.664 | 113.888 | *Luffa acutangula* | 8 | 5♀+3♂ | 0/8 |
| *Liriomyza sativae* | 2016.07 | Dongguan, Guangdong, China | 22.923 | 113.899 | *Vigna sinensis* | 6 | 3♀+3♂ | 0/6 |
| *Liriomyza sativae* | 2017.12 | Heyuan, Guangdong, China | 23.716 | 114.681 | *Solanum lycopersicum* | 4 | 2♀+2♂ | 0/4 |
| *Liriomyza sativae* | 2016.08 | Dongying, Shandong, China | 37.564 | 118.246 | *Cucurbita moschata* | 8 | 3♀+5♂ | 0/8 |
| *Liriomyza sativae* | 2016.09 | Yantai, Shandong, China | 37.504 | 121.369 | *Phaseolus vulgaris* | 4 | 2♀+2♂ | 0/4 |
| *Liriomyza sativae* | 2019.09 | Chiayi, Taiwan, China | 23.400 | 120.551 | *Luffa cylindrica* | 6 | 3♀+3♂ | 0/6 |
| *Liriomyza sativae* | 2019.09 | Tainan, Taiwan, China | 23.283 | 120.447 | *Luffa cylindrica* | 6 | 3♀+3♂ | 0/6 |
| *Liriomyza sativae* | 2019.05 | Keaau, Hawaii, USA | 19.629 | -155.027 | *Solanum nigrum* | 2 | 1♀+1♂ | 0/2 |
| *Liriomyza sativae* | 2019.05 | Paradise Park, Hawaii, USA | 19.588 | -154.96 | *Solanum melongena* | 11 | 6♀+5♂ | 0/11 |
| *Liriomyza sativae* | 2019.05 | USDA-ARS-PBARC, Hawaii, USA | 19.698 | -155.093 | *Solanum melongena* | 9 | 5♀+4♂ | 0/9 |
| *Liriomyza sativae* | 2019.05 | Kawamata, Hawaii, USA | 20.011 | -155.684 | *Solanum melongena* | 5 | 3♀+2♂ | 0/5 |
| *Liriomyza sativae* | 2019.05 | Hilo, Hawaii, USA | 19.671 | -155.100 | *Solanum melongena* | 5 | 4♀+1♂ | 0/5 |
| *Liriomyza sativae* | 2017.07 | Vero Beach, Florida, USA | 28.374 | -81.549 | Laboratory colony | 20 | 10♀+10♂ | 20/0 |
| *Liriomyza sativae* | 2020.01 | Kirinyaga County, Kenya | -0.615 | 37.375 | *Solanum lycopersicum* | 23 | 11♀+12♂ | 0/23 |
| *Liriomyza huidobrensis* | 2019.04 | Bali, Indonesia | -8.340 | 115.091 | *Brassica rapa* | 12 | 6♀+6♂ | 12/0 |

| | | | | | | | | |
|---|---|---|---|---|---|---|---|---|
| *Liriomyza huidobrensis* | 2020.01 | Nairobi County, Kenya | -1.220 | 36.895 | *Vicia faba* | 10 | 5♀+5♂ | 10/0 |
| *Liriomyza huidobrensis* | 2020.12 | Tarome, QLD, Australia | -27.988 | 152.475 | *Apium graveolens* | 17 | larvae | 17/0 |
| *Liriomyza huidobrensis* | 2020.12 | Kalabar, QLD, Australia | -27.938 | 152.588 | *Phaseolus vulgaris* | 6 | larvae | 6/0 |
| *Liriomyza trifolii* | 2019.04 | Bali, Indonesia | -8.340 | 115.091 | *Chrysanthemum* | 12 | 6♀+6♂ | 0/12 |
| *Liriomyza trifolii* | 2017.07 | California, USA | 33.974 | -117.327 | Laboratory colony | 20 | 10♀+10♂ | 20/0 |
| *Liriomyza trifolii* | 2020.02 | Kirinyaga County, Kenya | -0.615 | 37.375 | *Solanum lycopersicum* | 1 | 1♀ | 1/0 |
| *Liriomyza trifolii* | 2020.02 | Qereqere, Sigatoka Valley, Fiji | -18.051 | 177.559 | *Vigna unguiculata* ssp. *sesquipedalis* | 12 | larvae | 12/0 |
| *Liriomyza trifolii* | 2020.02 | Wainibokasi, Nausori, Fiji | -18.060 | 178.572 | *Vigna unguiculata* ssp. *sesquipedalis* | 11 | larvae | 11/0 |
| *Liriomyza trifolii* | 2020.02 | Koronivia, Nausori, Fiji | -18.049 | 178.541 | *Vigna unguiculata* ssp. *sesquipedalis* | 4 | larvae | 4/0 |
| *Liriomyza trifolii* | 2017.09 | Bazartete, Leoreka, Timor-Leste | -8.677 | 125.372 | *Chrysanthemum* sweeping | 3 | adult | 3/0 |
| *Liriomyza bryoniae* | 2020.07 | Berkel en Rodenrijs, Netherlands | 51.991 | 4.473 | Laboratory colony | 20 | 10♀+10♂ | 20/0 |
| *Liriomyza chinensis* | 2019.04 | Bali, Indonesia | -8.324 | 115.169 | *Allium fistulosum.* | 12 | 6♀+6♂ | 12/0 |
| *Liriomyza brassicae* | 2019.12 | Flemington Bridge, VIC, Australia | -37.787 | 144.939 | *Brassica fruticulosa* | 192 | 96♀+96♂ | 192/0 |
| *Liriomyza brassicae* | 2019.01 | Gladstone Park, VIC, Australia | -37.692 | 144.895 | *Brassica fruticulosa* | 6 | 3♀+3♂ | 6/0 |
| *Liriomyza brassicae* | 2018.01 | Northcote, VIC, Australia | -37.769 | 144.999 | *Brassica fruticulosa* | 12 | 6♀+6♂ | 12/0 |

| | | | | | | | | |
|---|---|---|---|---|---|---|---|---|
| *Liriomyza brassicae* | 2019.12 | Fitzroy North, VIC, Australia | -37.783 | 144.993 | *Brassica fruticulosa* | 24 | 12♀+12♂ | 24/0 |
| *Liriomyza brassicae* | 2018.12 | Thomastown, VIC, Australia | -37.685 | 145.013 | *Brassica fruticulosa* | 6 | 3♀+3♂ | 6/0 |
| *Liriomyza brassicae* | 2018.12 | Werribee VIC, Australia | -37.913 | 144.669 | *Brassica fruticulosa* | 24 | 12♀+12♂ | 24/0 |
| *Liriomyza brassicae* | 2018.12 | Werribee South, VIC, Australia | -37.971 | 144.700 | *Cakile maritima* | 8 | 3♀+5♂ | 8/0 |
| *Liriomyza brassicae* | 2018.12 | Bruce, ACT, Australia | -35.238 | 149.085 | *Brassica fruticulosa* | 24 | 12♀+12♂ | 24/0 |
| *Liriomyza brassicae* | 2019.09 | Lesmurdie, WA, Australia | -31.992 | 116.040 | *Tropaeolum majus* | 24 | 12♀+12♂ | 24/0 |
| *Liriomyza brassicae* | 2018.11 | Seloi Malere, Timor-Leste | -8.704 | 125.609 | Swept from *Cucurbita* crop | 21 | 16♀+5 ♂ | 21/0 |
| *Liriomyza brassicae* | 2017.09 | Seloi Kraik, Timor-Leste | -8.698 | 125.534 | Swept from *Cucurbita* crop | 1 | 1♀ | 1/0 |
| *Liriomyza chenopodii* | 2019.11 | Flemington Bridge, VIC, Australia | -37.787 | 144.939 | *Stellaria media* | 24 | 12♀+12♂ | 24/0 |
| *Liriomyza chenopodii* | 2019.11 | Werribee South, VIC, Australia | -37.966 | 144.685 | *Stellaria media* | 24 | 12♀+12♂ | 24/0 |
| *Liriomyza chenopodii* | 2019.11 | Glen Waverley, VIC, Australia | -37.882 | 145.165 | *Stellaria media* | 24 | 12♀+12♂ | 24/0 |
| *Liriomyza chenopodii* | 2019.11 | Fitzroy North, VIC, Australia | -37.783 | 144.993 | *Stellaria media* | 24 | 12♀+12♂ | 24/0 |
| *Liriomyza chenopodii* | 2019.11 | Werribee, VIC, Australia | -37.913 | 144.669 | *Stellaria media* | 24 | 12♀+12♂ | 24/0 |
| *Phytomyza plantaginis* | 2019.01 | Flemington Bridge, VIC, Australia | -37.787 | 144.939 | *Plantago major* | 25 | 13♀+12♂ | 25/0 |
| *Phytomyza plantaginis* | 2019.01 | Flemington Bridge, VIC, Australia | -37.787 | 144.939 | *Plantago lanceolata* | 35 | 18♀+17♂ | 35/0 |
| *Phytomyza plantaginis* | 2019.06 | Glenrowan, VIC, Australia | -36.461 | 146.224 | *Plantago lanceolata* | 24 | 24♀ | 24/0 |

| | | | | | | | | |
|---|---|---|---|---|---|---|---|---|
| *Phytomyza plantaginis* | 2019.06 | Stanhope, VIC, Australia | -36.446 | 144.982 | *Plantago lanceolata* | 24 | 24♀ | 24/0 |
| *Phytomyza plantaginis* | 2019.06 | Elmore, VIC, Australia | -36.493 | 144.608 | *Plantago lanceolata* | 4 | 4♀ | 4/0 |
| *Phytomyza plantaginis* | 2019.06 | Romsey, VIC, Australia | -37.354 | 144.747 | *Plantago lanceolata* | 5 | 5♀ | 5/0 |
| *Phytomyza plantaginis* | 2018.06 | Lismore, NSW, Australia | -28.809 | 153.288 | *Plantago lanceolata* | 24 | 24♀ | 24/0 |
| *Phytomyza plantaginis* | 2018.12 | Bruce, ACT, Australia | -35.238 | 149.085 | *Plantago lanceolata* | 32 | 32♀ | 32/0 |
| *Phytomyza syngenesiae* | 2019.08 | Flemington Bridge, VIC, Australia | -37.787 | 144.939 | *Sonchus oleraceus* | 192 | 96♀+96♂ | 192/0 |
| *Phytomyza syngenesiae* | 2019.08 | Werribee South, VIC, Australia | -37.966 | 144.685 | *Sonchus oleraceus* | 24 | 12♀+12♂ | 24/0 |
| *Phytomyza syngenesiae* | 2019.08 | Glen Waverley, VIC, Australia | -37.882 | 145.165 | *Sonchus oleraceus* | 24 | 12♀+12♂ | 24/0 |
| *Phytomyza syngenesiae* | 2019.08 | Fitzroy North, VIC, Australia | -37.783 | 144.993 | *Sonchus oleraceus* | 24 | 12♀+12♂ | 24/0 |
| *Phytomyza syngenesiae* | 2019.08 | Werribee, VIC, Australia | -37.913 | 144.669 | *Sonchus oleraceus* | 24 | 12♀+12♂ | 24/0 |
| *Phytomyza syngenesiae* | 2018.12 | Bruce, ACT, Australia | -35.238 | 149.085 | *Sonchus oleraceus* | 24 | 12♀+12♂ | 24/0 |
| *Phytomyza syngenesiae* | 2019.12 | Yanakie, VIC, Australia | -38.743 | 146.135 | *Sonchus oleraceus* | 8 | 4♀+4♂ | 8/0 |
| *Phytomyza syngenesiae* | 2019.09 | Lesmurdie, WA, Australia | -31.992 | 116.040 | *Sonchus oleraceus* | 24 | 12♀+12♂ | 24/0 |
| *Phytomyza syngenesiae* | 2019.08 | Ballina, NSW, Australia | -28.837 | 153.562 | *Sonchus oleraceus* | 16 | 8♀+8♂ | 16/0 |
| *Phytoliriomyza praecellens* | 2019.11 | Royal Park, VIC, Australia | -37.795 | 144.949 | *Rhagodia parabolica* | 8 | 4♀+4♂ | 8/0 |
| *Cerodontha milleri* | 2019.12 | Parkville, VIC, Australia | -37.800 | 144.956 | Unknown grass | 12 | 6♀+6♂ | 0/12 |

| | | | | | | | | |
|---|---|---|---|---|---|---|---|---|
| *Cerodontha milleri* | 2019.12 | Yanakie, VIC, Australia | -38.743 | 146.135 | Unknown grass | 12 | 6♀+6♂ | 0/12 |
| *Scaptomyza australis* | 2019.11 | Flemington Bridge, VIC, Australia | -37.787 | 144.939 | *Stellaria media* | 24 | 12♀+12♂ | 24/0 |
| *Scaptomyza australis* | 2019.11 | Werribee South, VIC, Australia | -37.966 | 144.685 | *Stellaria media* | 24 | 12♀+12♂ | 24/0 |
| *Scaptomyza australis* | 2019.11 | Fitzroy North, VIC, Australia | -37.783 | 144.993 | *Stellaria media* | 24 | 12♀+12♂ | 24/0 |
| *Scaptomyza flava* | 2019.12 | Flemington Bridge, VIC, Australia | -37.787 | 144.939 | *Brassica fruticulosa* | 6 | 3♀+3♂ | 6/0 |
| *Scaptomyza flava* | 2019.11 | Shoreham, VIC, Australia | -38.427 | 145.050 | *Tropaeolum majus* | 6 | 3♀+3♂ | 6/0 |

QLD = Queensland, ACT = Australian Capital Territory, NSW = New South Wales, VIC = Victoria, WA = Western Australia.

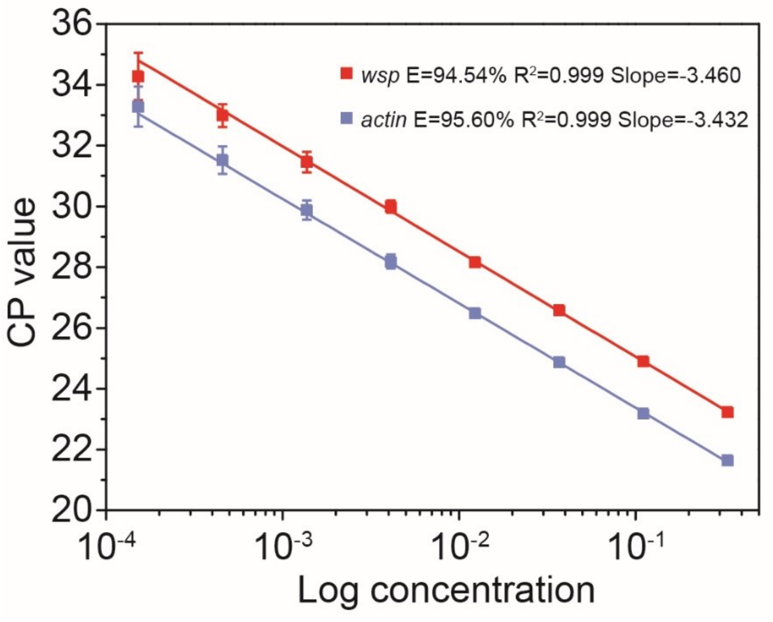


**Figure S1.** Estimation of qPCR amplification efficiencies of *actin* and *wsp* of *Liriomyza brassicae*. The original DNA concentration was 1 ng/μL. Standard curves of qPCR used threefold serial dilutions (10μL of dilution added to 20 μL of $H_2O$) of *L. brassicae* DNA on three separate occasions under identical conditions.

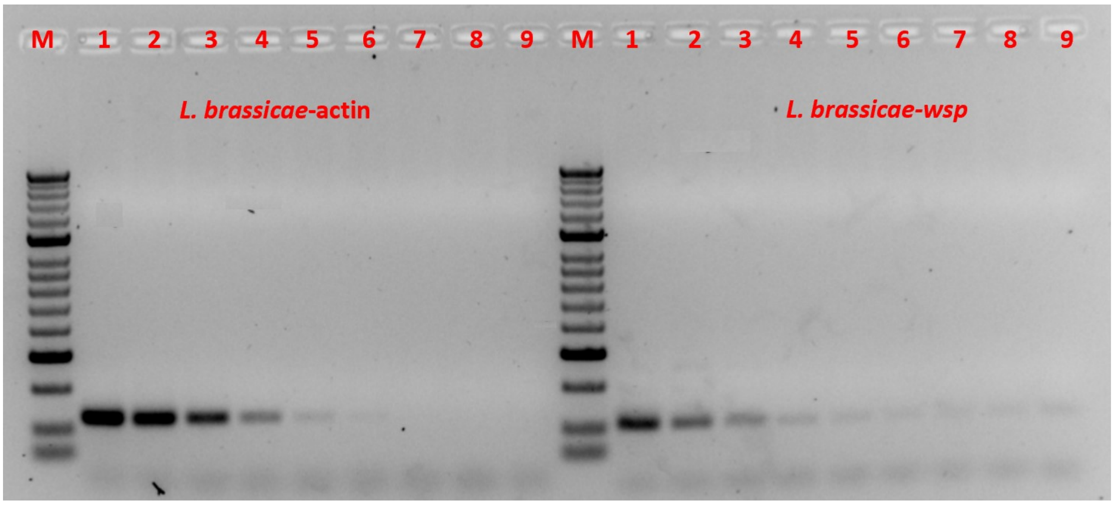


**Figure S2.** Agarose electrophoresis gel of conventional PCR (*actin* and *wsp*) of *Liriomyza brassicae*. Lanes 1-8: DNA diluted from $3^{-1}$ to $3^{-8}$ (threefold serial dilutions), Lane 9: negative control (water).

**Table S2.** Specific primers used for quantitative PCR and DNA barcoding of the leafminer species and *Wolbachia*. For other species, universal primers from Baldo et al. [1] were used.

| **Gene** | **Species** | **Primer sequence (5′→3）** |
|---|---|---|
| Primers used in quantitative PCR | | |
| *actin* | *L. brassicae* | TGACTGAAGCCCCCTTGAAC |
| | | GACCGGAAGCGTACAGTGAC |
| *wsp* | *L. brassicae* | TGTTGCAGACAGTTTAACAGCA |
| | | CACCAACACCAACACCAACG |
| Primers used in conventional PCR | | |
| *wsp* | *P. plantaginis* | CTAGCTACTACGTTCGTTTACAA |
| | | AAAACTAGCACCATAAGAACC |
| *wsp* | *P. syngenesiae* | TGATGAGGAAACTAGCTACT |
| | | AGCACCATAAGAACCAAAAT |

**Table S3.** Taxonomic information of leaf-mining flies and *Wolbachia wsp* sequences. Allele names correspond to the taxonomic placement of the host species.

| **Family** | **Genus** | **Species** | **Country** | **Location** | ***Wolbachia* supergroup** | ***Wolbachia wsp* allele** | **GenBank accession** |
|---|---|---|---|---|---|---|---|
| Agromyzidae | *Liriomyza* | *sativae* | Timor-Leste | Seloi Malere, Aileu | B | *w*LsatA | MW047079 |
| Agromyzidae | *Liriomyza* | *sativae* | Timor-Leste | Seloi Kraik, Alieu | B | *w*LsatB | MW310410 |
| Agromyzidae | *Liriomyza* | *sativae* | Timor-Leste | Liquisa, Dato | B | *w*LsatA | MW047079 |
| Agromyzidae | *Liriomyza* | *sativae* | Timor-Leste | Ermera, Mertutu | B | *w*LsatA | MW047079 |
| Agromyzidae | *Liriomyza* | *sativae* | Vietnam | Sao Bay | A | *w*LsatC | MW310402 |
| Agromyzidae | *Liriomyza* | *sativae* | Australia | Thursday Island | B | *w*LsatD | MW310403 |
| Agromyzidae | *Liriomyza* | *trifolii* | USA | California | B | *w*LsatD | MW310404 |
| Agromyzidae | *Liriomyza* | *trifolii* | Kenya | Kirinyaga County | B | *w*LtriA | MW310405 |
| Agromyzidae | *Liriomyza* | *trifolii* | Timor-Leste | Bazartete, Leoreka | B | *w*LsatA | MZ846189 |
| Agromyzidae | *Liriomyza* | *trifolii* | Japan | Shizuoka, Hamamatsu | B | *w*LsatD | AB231466 |
| Agromyzidae | *Liriomyza* | *trifolii* | Japan | Shizuoka, Hamamatsu | B | *w*LsatD | AB231467 |
| Agromyzidae | *Liriomyza* | *trifolii* | Japan | Miyagi | B | *w*LsatD | AB231468 |
| Agromyzidae | *Liriomyza* | *trifolii* | Fiji | Qereqere, Sigatoka Valley | B | *w*LsatD | MZ423195 |
| Agromyzidae | *Liriomyza* | *trifolii* | Fiji | Wainibokasi, Nausori | B | *w*LsatD | MZ423196 |
| Agromyzidae | *Liriomyza* | *trifolii* | Fiji | Koronivia, Nausori | B | *w*LsatD | MZ423197 |
| Agromyzidae | *Liriomyza* | *huidobrensis* | Indonesia | Bali | A | *w*LhuiA | MW052256 |
| Agromyzidae | *Liriomyza* | *huidobrensis* | Kenya | Nairobi County | A | *w*LhuiA | MW052255 |
| Agromyzidae | *Liriomyza* | *huidobrensis* | Australia | Tarome, QLD | B | *w*LsatA | MW491889 |
| Agromyzidae | *Liriomyza* | *huidobrensis* | Australia | Kalabar, QLD | B | *w*LsatA | MW491889 |
| Agromyzidae | *Liriomyza* | *bryoniae* | Netherlands | Berkel en Rodenrijs | B | *w*LbryA | MW310409 |

| Family | Genus | Species | Country | Location | *Wolbachia* supergroup | *Wolbachia wsp* allele | GenBank accession |
|---|---|---|---|---|---|---|---|
| Agromyzidae | *Liriomyza* | *bryoniae* | Japan | Hamamatsu, Shizuoka | B | *w*LbryB | AB231469.1 |
| Agromyzidae | *Liriomyza* | *chinensis* | Indonesia | Tabanan Regency, Bali | B | *w*LchiA | MW310406 |
| Agromyzidae | *Liriomyza* | *brassicae* | Timor-Leste | Seloi Malere | B | *w*LsatA | MW047078 |
| Agromyzidae | *Liriomyza* | *brassicae* | Timor-Leste | Seloi Kraik | B | *w*LsatA | MW047078 |
| Agromyzidae | *Liriomyza* | *brassicae* | Australia | Flemington Bridge, VIC | B | *w*LsatA | MW047082 |
| Agromyzidae | *Liriomyza* | *brassicae* | Australia | Gladstone Park, VIC | B | *w*LsatA | MW047082 |
| Agromyzidae | *Liriomyza* | *brassicae* | Australia | Northcote, VIC | B | *w*LsatA | MW047082 |
| Agromyzidae | *Liriomyza* | *brassicae* | Australia | Fitzroy North, VIC | B | *w*LsatA | MW047082 |
| Agromyzidae | *Liriomyza* | *brassicae* | Australia | Thomastown, VIC | B | *w*LsatA | MW047082 |
| Agromyzidae | *Liriomyza* | *brassicae* | Australia | Werribee, VIC | B | *w*LsatA | MW047082 |
| Agromyzidae | *Liriomyza* | *brassicae* | Australia | Werribee South, VIC | B | *w*LsatA | MW047082 |
| Agromyzidae | *Liriomyza* | *brassicae* | Australia | Bruce, ACT | B | *w*LsatA | MW047082 |
| Agromyzidae | *Liriomyza* | *brassicae* | Australia | Lesmurdie, WA | B | *w*LsatA | MW047082 |
| Agromyzidae | *Liriomyza* | *chenopodii* | Australia | Flemington Bridge, VIC | B | *w*LsatA | MW047081 |
| Agromyzidae | *Liriomyza* | *chenopodii* | Australia | Werribee South, VIC | B | *w*LsatA | MW047081 |
| Agromyzidae | *Liriomyza* | *chenopodii* | Australia | Glen Waverley, VIC | B | *w*LsatA | MW047081 |
| Agromyzidae | *Liriomyza* | *chenopodii* | Australia | Fitzroy North, VIC | B | *w*LsatA | MW047081 |
| Agromyzidae | *Liriomyza* | *chenopodii* | Australia | Werribee, VIC | B | *w*LsatA | MW047081 |
| Agromyzidae | *Phytomyza* | *plantaginis* | Australia | Flemington Bridge, VIC | B | *w*LsatA | MW310407 |
| Agromyzidae | *Phytomyza* | *plantaginis* | Australia | Flemington Bridge, VIC | B | *w*LsatA | MW310407 |
| Agromyzidae | *Phytomyza* | *plantaginis* | Australia | Glenrowan, VIC | B | *w*LsatA | MW310407 |

| Family | Genus | Species | Country | Location | *Wolbachia* supergroup | *Wolbachia wsp* allele | GenBank accession |
|---|---|---|---|---|---|---|---|
| Agromyzidae | *Phytomyza* | *plantaginis* | Australia | Stanhope, VIC | B | *w*LsatA | MW310407 |
| Agromyzidae | *Phytomyza* | *plantaginis* | Australia | Elmore, VIC | B | *w*LsatA | MW310407 |
| Agromyzidae | *Phytomyza* | *plantaginis* | Australia | Romsey, VIC | B | *w*LsatA | MW310407 |
| Agromyzidae | *Phytomyza* | *plantaginis* | Australia | Lismore, NSW | B | *w*LsatA | MW310407 |
| Agromyzidae | *Phytomyza* | *plantaginis* | Australia | Bruce, ACT | B | *w*LsatA | MW310407 |
| Agromyzidae | *Phytomyza* | *syngenesiae* | Australia | Flemington Bridge, VIC | A/B | *w*LsatC/*w*LsatA | MW471003/MW047083 |
| Agromyzidae | *Phytomyza* | *syngenesiae* | Australia | Werribee South, VIC | B | *w*LsatA | MW047083 |
| Agromyzidae | *Phytomyza* | *syngenesiae* | Australia | Glen Waverley, VIC | B | *w*LsatA | MW047083 |
| Agromyzidae | *Phytomyza* | *syngenesiae* | Australia | Fitzroy North, VIC | B | *w*LsatA | MW047083 |
| Agromyzidae | *Phytomyza* | *syngenesiae* | Australia | Werribee VIC | B | *w*LsatA | MW047083 |
| Agromyzidae | *Phytomyza* | *syngenesiae* | Australia | Bruce, ACT | B | *w*LsatA | MW047083 |
| Agromyzidae | *Phytomyza* | *syngenesiae* | Australia | Yanakie, VIC | B | *w*LsatA | MW047083 |
| Agromyzidae | *Phytomyza* | *syngenesiae* | Australia | Lesmurdie, WA | B | *w*LsatA | MW047083 |
| Agromyzidae | *Phytomyza* | *syngenesiae* | Australia | Ballina, NSW | B | *w*LsatA | MW047083 |
| Agromyzidae | *Phytoliriomyza* | *praecellens* | Australia | Royal Park, VIC | A | *w*LsatC | MW310408 |
| Drosophilidae | *Scaptomyza* | *flava* | Australia | Flemington Bridge, VIC | A | *w*LhuiA | MW052254 |
| Drosophilidae | *Scaptomyza* | *flava* | Australia | Shoreham, VIC | A | *w*LhuiA | MW052254 |
| Drosophilidae | *Scaptomyza* | *australis* | Australia | Flemington Bridge, VIC | B | *w*LsatA | MZ571835 |
| Drosophilidae | *Scaptomyza* | *australis* | Australia | Werribee South, VIC | B | *w*LsatA | MZ571835 |
| Drosophilidae | *Scaptomyza* | *australis* | Australia | Fitzroy North, VIC | B | *w*LsatA | MZ571835 |

QLD = Queensland, ACT = Australian Capital Territory, NSW = New South Wales, VIC = Victoria, WA = Western Australia.

**References**

1. Baldo, L.; Dunning Hotopp, J.C.; Jolley, K.A.; Bordenstein, S.R.; Biber, S.A.; Choudhury, R.R.; Hayashi, C.; Maiden, M.C.; Tettelin, H.; Werren, J.H. Multilocus sequence typing system for the endosymbiont *Wolbachia pipientis*. *Appl. Environ. Microbiol.* **2006**, *72*, 7098–7110.
